# Anatomically induced changes in rice leaf mesophyll conductance explain the variation in photosynthetic nitrogen use efficiency under contrasting nitrogen supply

**DOI:** 10.1186/s12870-020-02731-7

**Published:** 2020-11-18

**Authors:** Limin Gao, Zhifeng Lu, Lei Ding, Kailiu Xie, Min Wang, Ning Ling, Shiwei Guo

**Affiliations:** Jiangsu Provincial Key Lab for Organic Solid Waste Utilization, National Engineering Research Center for Organic-based Fertilizers, Jiangsu Collaborative Innovation Center for Solid Organic Waste Resource Utilization, Nanjing Agricultural University, Weigang 1, Nanjing, 210095 China

**Keywords:** Leaf anatomies, NH_4_^+^, NO_3_^−^, Mesophyll conductance, PNUE, Rubisco

## Abstract

**Background:**

The ratio of CO_2_ mesophyll conductance (*g*_m_) to Ribulose-1, 5-bisphosphate carboxylase/oxygenase (Rubisco) content has been suggested to positively affect photosynthetic nitrogen use efficiency (PNUE). The anatomical basis of *g*_m_ has been quantified, but information on the relationship between cell-level anatomies and PNUE is less advanced. Here, hydroponic experiments were conducted in rice plants supplied with ammonium (NH_4_^+^) and nitrate (NO_3_^−^) under three N levels (low, 0.71 mM; intermediate, 2.86 mM; high, 7.14 mM) to investigate the gas exchange parameters, leaf anatomical structure and PNUE.

**Results:**

The results showed a lower PNUE in plants supplied with high nitrogen and NH_4_^+^, which was positively correlated with the *g*_m_/Rubisco ratio. A one-dimensional within-leaf model revealed that the resistance to CO_2_ diffusion in the liquid phase (*r*_liq_) dominated the overall mesophyll resistance (*r*_m_), in which CO_2_ transfer resistance in the cell wall, cytoplasm and stroma were significantly affected by nitrogen supply. The chloroplast surface area exposed to intercellular space (*S*_c_) per Rubisco rather than the *g*_m_/*S*_c_ ratio was positively correlated with PNUE and was thus considered a key component influencing PNUE.

**Conclusion:**

In conclusion, our study emphasized that *S*_c_ was the most important anatomical trait in coordinating *g*_m_ and PNUE with contrasting N supply.

**Supplementary Information:**

The online version contains supplementary material available at 10.1186/s12870-020-02731-7.

## Background

Photosynthetic nitrogen use efficiency (PNUE), determined as the ratio of photosynthesis rate (*P*_n_) to leaf organic nitrogen content [1], is a key component of nitrogen use efficiency (NUE) and an indicator of the relationship between leaf nitrogen (N) and *P*_n_. Under the present atmosphere, the unsaturated CO_2_ concentration in C3 leaves influences the carboxylation of Ribulose-1,5-disphosphate (RuBP) and results in a finite *P*_n_, which fails to match the increase in leaf N and induces a decrease in PNUE [2]. By using an “evolutionary” algorithm, the partitioning of photosynthetic enzymes was altered based on a fixed total amount of protein-nitrogen for maximizing *P*_n_, and the result showed that an increase in Ribulose-1, 5-bisphosphate carboxylase/oxygenase (Rubisco) was required to maximize *P*_n_ [3]. It was also well documented that higher leaf N allocation into Rubisco was linked with an enhancement in PNUE [4].

Numerous studies have clarified that the enhancement in Rubisco activity is another favorable candidate for improving RuBP carboxylation efficiency and *P*_n_ because of its poor catalytic ability under ambient conditions due to the low CO_2_ concentration and the low affinity for CO_2_ [1, 5]. As the substrate of Rubisco, CO_2_ concentration in the chloroplast (*C*_c_), which is determined by stomatal conductance (*g*_s_) and mesophyll conductance (*g*_m_), plays a dominant role in regulating Rubisco activity [6, 7]. It has been demonstrated that *g*_m_ induces 40% of the total decrease in CO_2_ concentration between the atmosphere and the carboxylation sites of Rubisco [8]. In a previous study, Li et al. [5] argued that an increase in *g*_m_ was not sufficient to meet the carboxylation demand of the increased Rubisco content and eventually resulted in a decreased PNUE. Therefore, it is speculated that factors affecting *g*_m_ would influence Rubisco activity and the relationship between *P*_n_ and leaf N content.

Evidence is now mounting that *g*_m_ is largely dependent on leaf anatomical characteristics, including leaf thickness, cell wall thickness and chloroplast morphology [9, 10]. Higher leaf density and thicker mesophyll cell walls contribute to a reduction in *g*_m_ [9, 11–14], and mesophyll and/or chloroplast surface areas exposed to the intercellular space, *S*_mes_ and *S*_c_, respectively, are positively correlated with *g*_m_ [15]. The overall importance of different anatomical traits in the restriction of *g*_m_ varies [16]. For gymnosperms, the strongest sources of *g*_m_ are cell wall and chloroplast thickness, variation in chloroplast shape and size, and *S*_c_ [9]. In lycophytes and bryophytes, the highest CO_2_ diffusive resistance is mainly driven by extremely high cell wall thickness and low *S*_c_ [17]. Even though the anatomical factors influencing *g*_m_ have been widely studied, the role of these anatomical factors in influencing PNUE and their relative contribution in rice plants are still largely unknown.

Leaf anatomy is remarkably influenced by N nutrition; for example, decreasing leaf thickness and smaller chloroplasts with no starch granules have been detected in nitrogen-deficient leaves, while high-N leaves have more large chloroplasts with well-developed grana, stroma lamellae and starch granules per mesophyll cell [5, 18–20]. For different nitrogen forms, increased leaf thickness and a doubling of chloroplast volume with a larger internal membrane length have been found in NH_4_^+^-fed plants compared with NO_3_^−^-fed plants [21–23]. In this study, we examine the responses of leaf anatomical characteristics, including leaf thickness, mesophyll cell size, chloroplast length and thickness, chloroplast number per mesophyll cell under NH_4_^+^ and NO_3_^−^ nutrition with different N levels; moreover, we discuss the implications for understanding leaf trait variation with changes in N nutrition along the PNUE. Our objectives of the present study were as follows: (1) to identify the response of PNUE and leaf anatomical traits upon NH_4_^+^ and NO_3_^−^ nutrition at different N levels; (2) to clarify the role of leaf anatomical factors in coordinating the *g*_m_ and PNUE under NH_4_^+^ and NO_3_^−^ nutrition supply; and (3) to investigate the most limiting fraction of leaf anatomy in determining PNUE under different N supply.

## Results

### Effects of nitrogen supply on rice photosynthetic nitrogen use efficiency (PNUE)

**Fig. 1:**
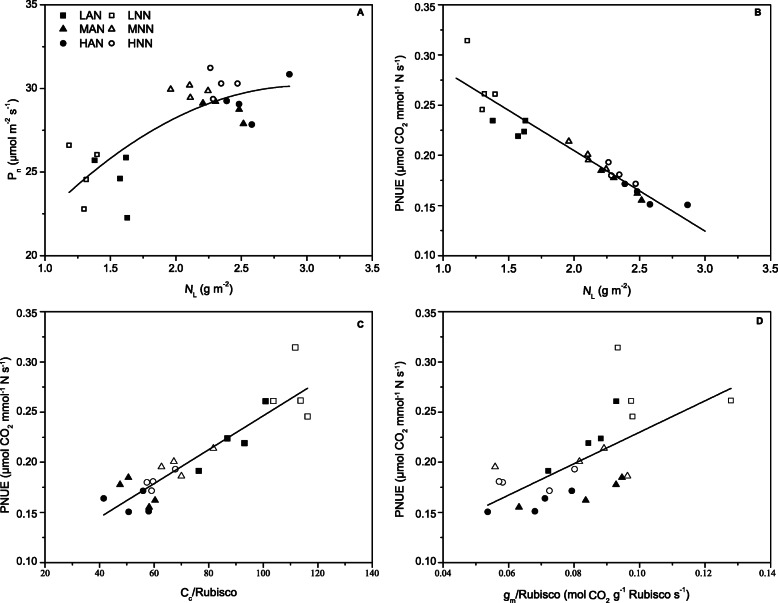
The relationship between leaf N content (*N*_L_) and the photosynthetic rate (*P*_n_) (**a**) and photosynthetic nitrogen use efficiency (PNUE) (**b**) and the relationship between PNUE and the ratio of chloroplast CO_2_ concentration to Rubisco (*C*_c_/Rubisco) (**c**) and the ratio of mesophyll conductance to Rubisco (*g*_m_/Rubisco) (**d**). Each point represents one replicate (four replicates per treatment). The lines represent the following regression equations: **a** y = − 1.8756 × ^2^ + 11.3310x + 13.0290, *R*^2^ = 0.6228, *P* < 0.05; **b** y = − 0.0763x + 0.3565, *R*^2^ = 0.7886, *P* < 0.05; **c** y = 0.0017x + 0.0774, *R*^2^ = 0.8295, *P* < 0.01; **d** y = 1.5663x + 0.0733, *R*^2^ = 0.4215, *P* < 0.01

Compared with those with low nitrogen supply (LAN and LNN), rice biomass and leaf area with intermediate and high nitrogen supply increased by 70–73% and 33–42% under NH_4_^+^ nutrition and by 30–48% and 40–41% under NO_3_^−^ nutrition, respectively (Table 1). There were no significant differences in rice biomass and leaf area between the intermediate and high nitrogen supply conditions. Rice biomass was less affected by nitrogen forms at the same nitrogen level, while for intermediate nitrogen supply, the leaf area was decreased by 10% in NO_3_^−^-fed plants than in NH_4_^+^-fed plants (Table 1). The leaf N content (*N*_L_) was 54–62% and 66–80% higher under intermediate N supply and high N supply than under low N supply and was decreased by 9–11% under NO_3_^−^ nutrition compared with that under NH_4_^+^ nutrition (Table 1). The Rubisco content was 26, 21 and 21% lower under low NO_3_^−^ (LNN), intermediate NO_3_^−^ (MNN) and high NO_3_^−^ (HNN) than that under low NH_4_^+^ (LAN), intermediate NH_4_^+^ (MAN) and high NH_4_^+^ (HAN), respectively (Table 1). The stomatal conductance (*g*_s_) was less affected by N supply than mesophyll conductance (*g*_m_), which was increased by 72 and 24% in HAN and HNN, respectively, compared with that in LAN and LNN and decreased by 4–30% under NO_3_^−^ compared to NH_4_^+^ nutrition (Table 1). Neither nitrogen supply levels nor nitrogen forms affected the chloroplast CO_2_ concentration (*C*_c_). With increasing leaf N content, the light-saturated photosynthetic rate (*P*_n_) increased, while the photosynthetic nitrogen use efficiency (PNUE) decreased (Table 1, Fig. 1a, b). The PNUE was 21, 17 and 14% higher in LNN, MNN and HNN than in LAN, MAN and HAN, respectively (Table 1). Positive correlations existed between PNUE and both the *C*_c_/Rubisco ratio and the *g*_m_/Rubisco ratio (Fig. 1c, d).

**Table 1:** Effects of different nitrogen supply levels on rice biomass (g), leaf area (cm^2^), leaf nitrogen content (*N*_L_, g m^− 2^), Rubisco content (g m^− 2^), stomatal conductance (*g*_s_, mol CO_2_ m^− 2^ s^− 1^), mesophyll conductance (*g*_m_, mol CO_2_ m^− 2^ s^− 1^), chloroplast CO_2_ concentration (*C*_c_, μmol mol^− 1^), photosynthesis rate (*P*_n_, μmol m^− 2^ s^− 1^), and photosynthetic nitrogen use efficiency (PNUE, μmol CO_2_ mmol^− 1^ N s^− 1^). Rice plants (“Zhendao 11”) were supplied with NH_4_^+^ (AN) or NO_3_^−^ (NN) under 3 different amounts, low N (0.71 mM, LAN and LNN), intermediate N (2.86 mM, MAN and MNN), and high N (7.14 mM, HAN and HNN). Data are presented as the means ± SD of four replications. Significant differences (*P* < 0.05) between treatments are indicated by different letters

Treatments	Biomass	Area	*N*_L_	Rubisco	*g*_s_	*g*_m_	*C*_c_	*P*_n_	PNUE
LAN	3.29 b	658 c	1.55 d	2.02 d	0.35 b	0.16 c	180 ab	24.61 b	0.22 b
MAN	5.71 a	932 a	2.38 b	3.16 b	0.45 a	0.24 ab	171 ab	28.74 a	0.17 cd
HAN	5.59 a	873 ab	2.58 a	3.67 a	0.49 a	0.28 a	187 a	29.25 a	0.16 d
LNN	3.77 b	595 c	1.30 e	1.49 e	0.27 b	0.16 c	165 b	25.00 b	0.27 a
MNN	4.92 a	837 b	2.11 c	2.50 c	0.44 a	0.20 bc	176 ab	29.86 a	0.20 bc
HNN	5.57 a	834 b	2.34 b	2.90 b	0.44 a	0.19 bc	176 ab	30.30 a	0.18 cd

### Effects of nitrogen supply on leaf anatomical properties

**Fig. 2:**
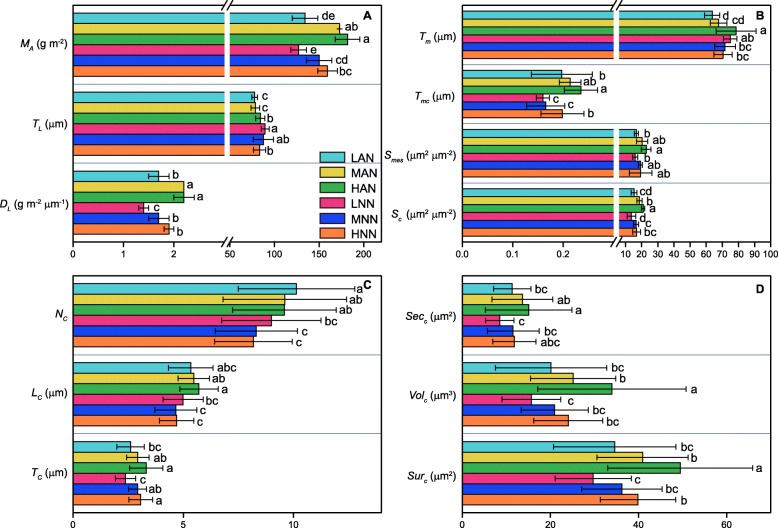
Effects of different nitrogen supply levels on integral leaf variables (**a**), mesophyll cell (**b**) and chloroplast (**c**, **d**) anatomical characteristics. *M*_A_ (g m^− 2^), leaf dry mass per area. *T*_L_ (μm), leaf thickness. *D*_L_ (g m^− 2^ μm^− 1^), leaf density. *T*_m_ (μm), mesophyll thickness. *T*_mc_ (μm), mesophyll cell wall thickness. *S*_mes_ (μm^2^ μm^− 2^), mesophyll surface area exposed to intercellular airspace. *S*_c_ (μm^2^ μm^− 2^), chloroplast surface area exposed to intercellular airspace. *N*_c_, chloroplast number per mesophyll cell. *L*_c_ (μm), chloroplast length. *T*_c_ (μm), chloroplast thickness. *Sec*_c_ (μm^2^), chloroplast section area. *Vol*_c_ (μm^3^), chloroplast volume. *Sur*_c_ (μm^2^), chloroplast surface area. The error bars indicate the standard deviation and at least 15 replicates were conducted for each parameter

With increasing leaf N supply levels, leaf thickness (*T*_L_) and mesophyll cell thickness (*T*_m_) increased in NH_4_^+^-fed plants but decreased in NO_3_^−^-fed plants (Supplementary Fig. S1, Fig. 2a, b). Leaf dry mass per area (*M*_A_), leaf density (*D*_L_) and mesophyll cell wall thickness (*T*_mc_) were increased by high N supply either with NH_4_^+^ or NO_3_^−^, and were lower under NO_3_^−^ nutrition than under NH_4_^+^ nutrition. Mesophyll surface area exposed to intercellular airspace (*S*_mes_) and the chloroplast surface area facing intercellular airspace (*S*_c_) were upregulated significantly by increasing the nitrogen supply level (Fig. 2b). The *S*_mes_ increased by 22–37 and 21% under intermediate and high N supply conditions in NH_4_^+^ and NO_3_^−^ nutrition, respectively, and the corresponding *S*_c_ increased by 22–38% and 21–24%, both compared with their respective low N supply conditions. No obvious differences in *S*_c_ between NH_4_^+^ and NO_3_^−^ under low N levels were observed, but the *S*_c_ decreased by 11 and 20% under MNN and HNN, respectively, compared to that under MAN and HAN (Fig. 2b).

We further analyzed the chloroplast number per mesophyll cell (*N*_c_), chloroplast length (*L*_c_), chloroplast thickness (*T*_c_), chloroplast surface area (*Sur*_c_), chloroplast volume (*Vol*_c_) and chloroplast section area (*Sec*_c_). *L*_c_ was lower in NO_3_^−^-fed plants than in NH_4_^+^-fed plants under intermediate and high N levels (Supplementary Fig. S1, Fig. 2c). Compared with that in LAN and LNN, the *T*_c_ was increased by 13–27% in MAN and HAN and 23–29% in MNN and HNN (Supplementary Fig. S1, Fig. 2c). Higher *Sur*_c_ and *Vol*_c_ were observed under high N supply. The chloroplast size was decreased under NO_3_^−^ nutrition by 10–22%, 15–33%, and 10–40% in *Sur*_c_, *Vol*_c_ and *Sec*_c_ under low N, intermediate N and high N supply, respectively (Supplementary Fig. S1, Fig. 2d).

### Anatomical limitations of mesophyll conductance

**Fig. 3:**
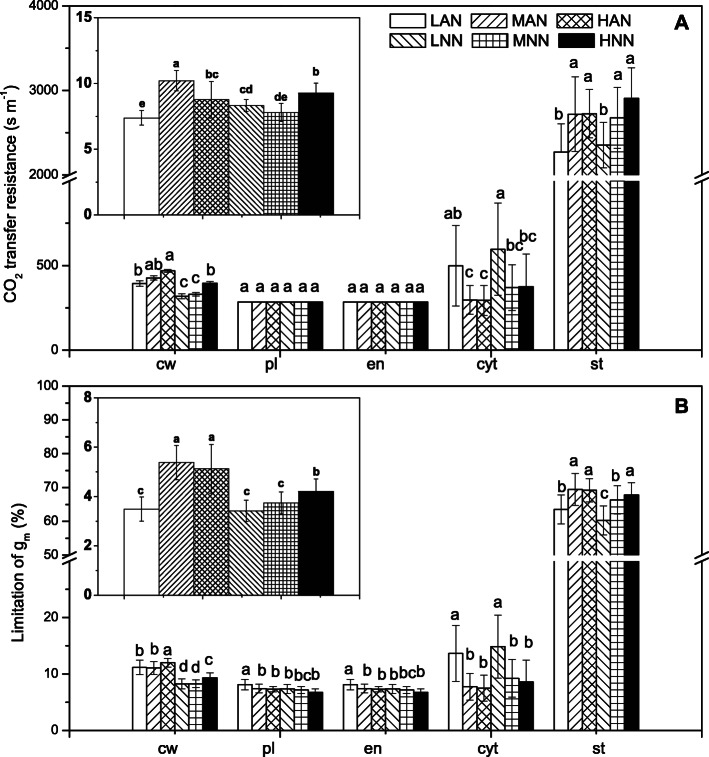
Anatomical limitations of mesophyll conductance (*g*_m_) (**a**) and the share of the overall *g*_m_ limitation (**b**) by cell wall (cw), plasma membrane (pl), chloroplast envelope (en), cytoplasm (cyt) and stroma (st) in rice leaves supplied with NH_4_^+^ (AN) or NO_3_^−^ (NN) under 3 different amounts, low N (0.71 mM, LAN and LNN), intermediate N (2.86 mM, MAN and MNN), and high N (7.14 mM, HAN and HNN). The inset figure shows the anatomical limitations of *g*_m_ and the share of the overall *g*_m_ limitation by gas phase. The error bars indicate the standard deviation and at least 15 replicates were conducted for each parameter. Different letters indicate statistically significant differences (*P* < 0.05) between different treatments

The values of *g*_m_ calculated according to the methods of Harley et al. [24] and Tomas et al. [16] were strongly positively linearly correlated (Supplementary Fig. S2, *R*^2^ = 0.936). Further quantitative analysis showed that both the resistance in the gas phase (*r*_ias_) and proportion of gas-phase limitations (*l*_ias_) of *g*_m_ had little impact on the overall mesophyll resistance (Fig. 3), and that the liquid phase resistance (*r*_liq_) was responsible for the limited *g*_m_ majority, among which stroma played a dominant role. High N supply significantly increased the resistance in the cell wall (*r*_cw_) and stroma (*r*_st_); compared with low N supply, *g*_m_ limited by the stroma (*l*_st_) was increased by 9–10% under moderate N supply and by 9–13% under high N supply (Fig. 3b). Consistent with the absolute cytoplasm resistance, *g*_m_ limited by the cytoplasm (*l*_cyt_) and cell wall (*l*_cw_) were downregulated under high N supply and NO_3_^−^ nutrition, respectively (Fig. 3). Among all the components, *r*_cw_ was the primary component affected by N forms and was 19, 23 and 16% higher under NH_4_^+^ nutrition than under NO_3_^−^ nutrition in low N, intermediate N and high N supply, respectively (Fig. 3a).

## Discussion

### Effects of N supply on the *g*_m_/Rubisco ratio and photosynthetic nitrogen use efficiency (PNUE)

Decreased PNUE under high N supply has been reported in previous and present studies (Table 1, Fig. 1) [5, 25–27]; referring to N forms, higher PNUE under NO_3_^−^ nutrition than NH_4_^+^ nutrition in the present study is consistent with results in barley (*Hordeum vulgare* L.) [28], pine [29], and cucumber [30]. Leaf nitrogen allocation is an important factor influencing PNUE. Onoda et al. [31] indicated that a higher fraction of photosynthetic nitrogen in electron transport and Rubisco would contribute to increased PNUE in leaves with lower dry mass per area (*M*_A_), while in leaves with higher *M*_A_, the over-investment of nitrogen in photosynthetic nitrogen and/or cell walls would reduce PNUE [1]. The effect of the proportion of Rubisco in leaf N content on PNUE can be expressed based on Eq. (6):

1 $$ \mathrm{PNUE}=\frac{g_{\mathrm{m}}}{\mathrm{Rubisco}}\left({C}_{\mathrm{i}}-{C}_{\mathrm{c}}\right)\frac{\mathrm{Rubisco}}{N_{\mathrm{L}}} $$

**Fig. 4:**
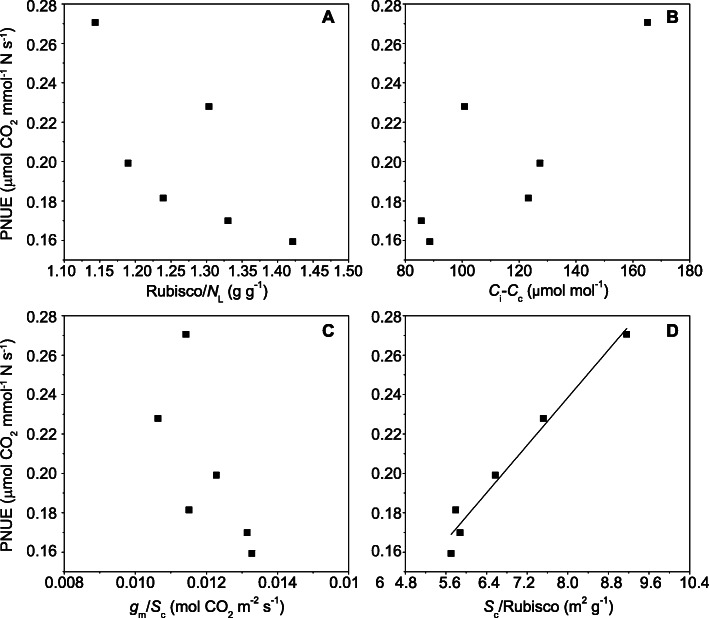
The relationship between photosynthetic nitrogen use efficiency (PNUE) and the ratio of Rubisco to leaf N content (Rubisco/*N*_L_) (**a**), the difference between intercellular CO_2_ concentration and chloroplast CO_2_ concentration (*C*_i_-*C*_c_) (**b**), the ratio of mesophyll conductance to chloroplast surface area exposed to intercellular airspace (*g*_m_/*S*_c_) (**c**), and the ratio of chloroplast surface area exposed to intercellular airspace to Rubisco (*S*_c_/Rubisco) (**d**). Data represent the mean of 4 replicates for PNUE, Rubisco, *N*_L_, *C*_i_, *C*_c_, *g*_m_ and at least 15 replicates for *S*_c_. The line in the figure represents the following regression equation: y = 0.0299x – 0.0019, *R*^2^ = 0.9700, *P* < 0.01

Our study detected that the Rubisco allocation ratio was increased under high nitrogen supply but decreased under NO_3_^−^ nutrition compared with NH_4_^+^ nutrition; however, the portion of Rubisco in leaf N content was not associated with PNUE (Fig. 4a). These results implied that Rubisco activity, rather than its content, played a dominant role in regulating PNUE [1, 5, 32].

An increased Rubisco allocation ratio requires increased CO_2_ partial pressure at the carboxylation site (*C*_c_) to meet carboxylation demands; however, the extent of the increase in *C*_c_ was less than that in Rubisco content, which resulted from the finite stomatal conductance (*g*_s_) and mesophyll conductance (*g*_m_). Li et al. [5] demonstrated that the smaller increases in *g*_m_ relative to Rubisco content resulted in relatively lower CO_2_ levels in chloroplasts and PNUE (Fig. 1c, d), which implied that the *g*_m_/Rubisco ratio rather than the absolute value of *g*_m_ was the key factor that regulates PNUE. We further compared the gap between estimated *g*_m_ and *C*_c_ proposed by Harley et al. [24] (Eq. 5, 6) and theoretical *C*_c_ (*C*_c-Theoretical_) and *g*_m_ (*g*_m-Theoretical_), which were calculated as follows based on Ding et al. [27], to evaluate the equilibrium state of *g*_m_ and Rubisco under different N nutrition conditions:

2 $$ {g}_{\mathrm{m}}-\mathrm{Theoretical}\ \left(\mathrm{intermediate}\ \mathrm{or}\ \mathrm{high}\ \mathrm{N}\right)=\mathrm{Rubisco}\ \left(\mathrm{intermediate}\ \mathrm{or}\ \mathrm{high}\ \mathrm{N}\right)\times \frac{g_{\mathrm{m}}}{\mathrm{Rubisco}}\left(\mathrm{low}\ \mathrm{N}\right) $$

3 $$ {C}_c-\mathrm{Theoretical}\ \left(\mathrm{intermediate}\ \mathrm{or}\ \mathrm{high}\ \mathrm{N}\right)=\mathrm{Rubisco}\ \left(\mathrm{intermediate}\ \mathrm{or}\ \mathrm{high}\ \mathrm{N}\right)\times \frac{C_{\mathrm{c}}}{\mathrm{Rubisco}}\ \left(\mathrm{low}\ \mathrm{N}\right) $$

**Fig. 5:**
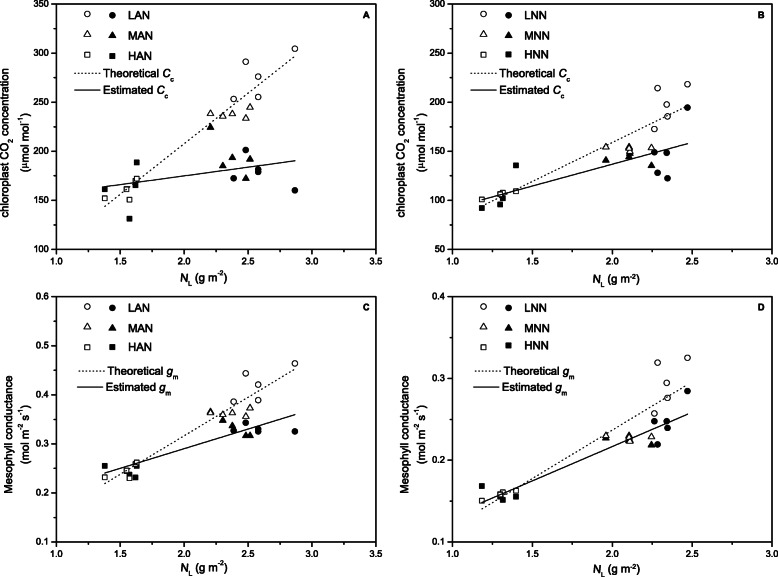
The relationships between the leaf-N content (*N*_L_) and the estimated and theoretical chloroplast CO_2_ concentration (*C*_c_) (**a**, **b**) and the estimated and theoretical mesophyll conductance (*g*_m_) (**c**, **d**) under NH_4_^+^ (**a**, **c**) and NO_3_^−^ (**b**, **d**) nutrition. Each point represents one replicate (four replicates per treatment). The dashed lines represent the theoretical chloroplast CO_2_ concentration and mesophyll conductance, which were calculated according to the ratio of *C*_c_/Rubisco and *g*_m_/Rubisco constant at low-N levels, and the theoretical *C*_c_ and *g*_m_ at intermediate and high N levels was Rubisco (intermediate or high N) × (*C*_c_/Rubisco or *g*_m_/Rubisco (low N)). The lines represent the estimated *C*_c_ and *g*_m_. Rice leaves supplied with NH_4_^+^ (AN) or NO_3_^−^ (NN) under 3 different amounts, low N (0.71 mM, LAN and LNN), intermediate N (2.86 mM, MAN and MNN), and high N (7.14 mM, HAN and HNN)

As shown in Fig. 5, both theoretical and estimated *C*_c_ and/or *g*_m_, as well as the differences between them, increased obviously with increasing leaf N content, and the gap between theoretical and estimated *C*_c_ and/or *g*_m_ under NH_4_^+^ nutrition was larger than that under NO_3_^−^ nutrition. These results confirmed that the balance between Rubisco content and *C*_c_ and/or *g*_m_ was weaker when high N and NH_4_^+^ were supplied, and the relatively lower *C*_c_ failed to meet the carboxylation demands of the increased Rubisco content, resulting in decreased PNUE (Fig. 1c, d).

### Overall importance of leaf anatomy in determining *g*_m_ and PNUE

When leaf nitrogen content was expressed on a leaf dry mass basis, no significant differences in leaf N content between NH_4_^+^ and NO_3_^−^ nutrition were obtained. Therefore, the discrepancies in PNUE between different N forms were primarily caused by the difference in *M*_A_ (Fig. 2), which resulted from leaf anatomy characteristics such as leaf density (*D*_L_), leaf thickness (*T*_L_) and cell wall thickness (*T*_mc_). In NO_3_^−^-fed plants, the lower *M*_A_ was the ultimate result of lower *D*_L_ and *T*_mc_. However, a lower *M*_A_ was not always related to a lower *g*_m_, as observed in the present study; Hassiotou et al. [8] found a negative relationship between *M*_A_ and *g*_m_ in the range of 100–500 g m^− 2^ of *M*_A_, while Hanba et al. [33] clarified a positive relationship in the same range of *M*_A_. These contrasting results were explained by different ways to enhance *M*_A_, as the increases in *D*_L_ and *T*_L_ were associated with higher *g*_m_, while the opposite conclusion would be obtained if the increase in *M*_A_ was a result of a thickened cell wall [8]. Our positive correlation between *M*_A_ and *g*_m_ implied that the contributions of *D*_L_ and/or *T*_L_ compensated for the inhibitory effect of *T*_mc_ on *g*_m_.

To qualify the relative importance of each leaf anatomy trait in explaining *g*_m_, a one-dimensional within-leaf model was calculated to clarify the limitation of *g*_m_ in each process [16]. The results showed that more than 90% of the total limitation of *g*_m_ came from *L*_liq_, which was a consequence of limitation in the cell wall (*L*_cw_), plasma membrane (*L*_pl_), envelope (*L*_en_), cytoplasm (*L*_cyt_), and stroma (*L*_st_) (Fig. 3) [34]. The decreased contribution of cytoplasmic resistance to *g*_m_ under high N resulted from the decreased distance between adjacent chloroplasts, rather than the distance between the cell wall and chloroplasts [16], and the increase in chloroplast thickness (*T*_c_) extended the transport path for CO_2_ from the chloroplast membrane to the carboxylation site in the interior of chloroplasts and resulted in an increasing in *r*_st_ [35, 36]. Except for the resistance of each part, the chloroplast surface area exposed to intercellular airspace (*S*_c_) was a paramount factor affecting CO_2_ liquid resistance (*r*_liq_). However, the decreased *r*_cyt_ and increased *S*_c_ partially compensated for the increased *r*_st_ under high N supply and resulted in an increased *g*_m_, although the effects were weak and did not match the increase in Rubisco content.

Considering the dominant role of *S*_c_ in determining *g*_m_, the *g*_m_/Rubisco in Eq. (1) was replaced by the product of *g*_m_/*S*_c_ and *S*_c_/Rubisco to demonstrate the effect of leaf anatomies and *g*_m_ on PNUE, which can be expressed as follows:

4 $$ \mathrm{PNUE}=\frac{g_{\mathrm{m}}}{S_{\mathrm{c}}}\ \frac{S_{\mathrm{c}}}{\mathrm{Rubisco}}\ \frac{\mathrm{Rubisco}}{N_{\mathrm{L}}}\left({C}_{\mathrm{i}}-{C}_{\mathrm{c}}\right) $$

According to the formula above and Terashima et al. [37], a positive correlation would be summarized between PNUE and *g*_m_/*S*_c_, which emphasized the potential role of leaf anatomical characteristics except for *S*_c_, as well as the activity of carbonic anhydrase (CA) in contributing to PNUE [37]. However, the weak negative relationship between *g*_m_/*S*_c_ and PNUE and the significant positive correlation between the *S*_c_/Rubisco ratio and PNUE suggested the dominant role of *S*_c_ in influencing PNUE. Due to the limited knowledge of the relationship between PNUE and the *S*_c_/Rubisco ratio, Onoda et al. [14] did not take this component into consideration when they analyzed the physiological and structural tradeoffs underlying the leaf economics spectrum, and they argued that *S*_c_ per Rubisco may not correlate strongly with *M*_A_ or PNUE. However, we detected that *S*_c_ per Rubisco was a critical parameter associated with PNUE in rice plants supplied with different N nutrition levels. Similar results were well documented in a review by Terashima et al. [15] and Terashima et al. [37], in which they speculated that, from the perspective of Rubisco and nitrogen use efficiency, thicker leaves with larger *S*_c_ were advantageous because the increased ratio of *S*_c_ to Rubisco would increase chloroplast CO_2_ concentration. The increased *S*_c_/Rubisco ratio in NO_3_^−^– and high N–fed plants partially resulted from the lower leaf density, which allowed more chloroplast surface area to be exposed to intercellular airspace (Fig. 2a).

## Conclusions

In conclusion, we demonstrated that PNUE is decreased in rice plants supplied with high N and ammonium nutrition, which results from unbalanced increases in *g*_m_ and Rubisco content. Nitrogen-induced variation in *g*_m_ is associated with leaf anatomical traits, especially chloroplast surface area exposed to intercellular airspace (*S*_c_). We further concluded that the *S*_c_/Rubisco ratio is directly related to the response of PNUE to N supply and that its increase is advantageous to the increase in PNUE.

## Methods

### Plant material and growth conditions

Rice seeds (*Oryza sativa* L., ssp. japonica inbred, cv. ‘Zhendao 11’) were purchased from Mingtian Seed Company (Nanjing, China), disinfected with 10% H_2_O_2_ for 30 min and germinated in 2.0 mM CaSO_4_ at 25 °C. The rice seedlings were transferred to 6 L rectangular containers (30 × 20 × 10 cm) when the seedlings developed 2.5 visible leaves, and one quarter-strength mixture of NH_4_^+^ and NO_3_^−^ nutrient solution (for composition, see below) was supplied. Three days later, the seedlings were transferred to a one half-strength nutrient solution. After 6 days, the seedlings were supplied with full-strength nutrient solution for 1 week, after which the seedlings were supplied with either (NH_4_)_2_SO_4_ (AN) or Ca (NO_3_)_2_ (NN) at three different N levels: low N (0.71 mM), intermediate N (2.86 mM), and high N (7.14 mM). Thus, six treatments were applied: LAN (low NH_4_^+^), MAN (intermediate NH_4_^+^), HAN (high NH_4_^+^), LNN (low NO_3_^−^), MNN (intermediate NO_3_^−^), and HNN (high NO_3_^−^). In addition, the macronutrients in the solution were as follows (mM): 0.32 P as KH_2_PO_4_, 1.02 K as K_2_SO_4_ and KH_2_PO_4_ and 1.65 Mg as MgSO_4_. The micronutrients were (μM) as follows: 35.8 Fe as Fe-EDTA, 9.10 Mn as MnCl_2_·4H_2_O, 0.52 Mo as (NH_4_)_6_Mo_7_O_24_·4H_2_O, 18.5 B as H_3_BO_3_, 0.15 Zn as ZnSO4·7H_2_O, 0.16 Cu as CuSO_4_·5H_2_O and 100 Si as Na_2_SiO_3_·9H_2_O. CaCl_2_ was added to the AN, LNN, and MNN solutions to adjust the Ca level to that of the HNN treatment. The nitrification inhibitor dicyandiamide (DCD) was added to each nutrient solution to prevent the oxidation of NH_4_^+^. The nutrient solutions were changed every 3 days, and the pH was adjusted to 5.50 ± 0.05 each day with 0.1 M HCl or NaOH. All the treatments were replicated 5 times with a completely randomized design. The temperature in the greenhouse was maintained at 30 °C during the day and 18 °C at night. Light was supplied by SON-TAGRO 400 W bulbs, and the distance between the light and the rice plants was approximately 60 cm. The light intensity was maintained at a minimum of 1000 μmol photons m^− 2^ s^− 1^ at the leaf level using a 14-h photoperiod.

### Measurement of biomass and leaf N content

After all the measurements were completed, plant dry weight was determined after oven-drying at 105 °C for 30 min and then at 70 °C to a constant weight. Pictures of the leaves used for the measurement of *P*_n_ were taken with a camera along with a benchmark to calibrate, and the leaf area was obtained by ImageJ Pro Plus, after which the leaves were dried and digested with H_2_SO_4_-H_2_O_2_ at 260–270 °C. The leaf N concentration was determined using a digital colorimeter (AutoAnalyzer 3; Bran+Luebbe).

### Gas exchange measurements

Twenty days after treatments, a Li–Cor 6400 infrared gas analyzer was used for the simultaneous measurement of light–saturated photosynthesis (*P*_n_) and chlorophyll fluorescence on the newly expanded leaves from 9:00 to 15:00. Leaf temperatures were 25 °C, the relative humidity was 45%, and photosynthetic photon flux density (PPFD) was 1500 μmol m^− 2^ s^− 1^ for all measurements. After equilibration to a steady state, *P*_n_ was recorded and the photosynthetic nitrogen use efficiency (PNUE) was calculated as the ratio of *P*_n_ to the leaf nitrogen content per leaf area. The fluorescence (*F*_s_) was also measured simultaneously, and a 0.8 s saturating pulse of light (approx. 8000 μmol m^− 2^ s^− 1^) was applied to measure the maximum fluorescence (*F*_m_′). The efficiency of photosystem II (*Φ*_PSII_) was calculated as *Φ*_PSII_ = 1- *F*_s_/*F*_m_′. The total electron transport rate (*J*_T_) was calculated as *J*_T_ = *Φ*_PSII_ × PPFD×α_leaf_ × β, where α_leaf_ and β were leaf absorption and the proportion of quanta absorbed by photosystem II, respectively. In this study, α_leaf_ was also assumed to be 0.85, and β was assumed to be 0.5 [38].

The following equations proposed by Harley et al. [24] were used to calculate the CO_2_ mesophyll conductance (*g*_m_) and chloroplast CO_2_ concentration (*C*_c_):

5 $$ {g}_{\mathrm{m}}=\frac{P_{\mathrm{n}}}{C_{\mathrm{i}}-\varGamma \ast \frac{J_{\mathrm{T}}+8\times \left({P}_{\mathrm{n}}+{R}_{\mathrm{d}}\right)}{J_{\mathrm{T}}-4\times \left({P}_{\mathrm{n}}+{R}_{\mathrm{d}}\right)}} $$

6 $$ {C}_{\mathrm{c}}={C}_{\mathrm{i}}-\frac{P_{\mathrm{n}}}{g_{\mathrm{m}}} $$

where *C*_i_ is the intercellular CO_2_ concentration, Γ* is the CO_2_ compensation point and *R*_d_ is the mitochondrial respiration rate in the light. In the present experiment, Γ* and *R*_d_ were measured on newly expanded leaves according to the method of Li et al. [39]. PPFDs in the cuvette were controlled as the series of 150, 300, and 600 μmol m^− 2^ s^− 1^. At each PPFD, the ambient CO_2_ concentration in the cuvette was adjusted as the series of 25, 50, 75 and 100 μmol CO_2_ mol^− 1^. Thirty minutes prior to initiating measurements, leaves were placed in the cuvette with a PPFD of 600 μmol photons m^− 2^ s^− 1^ and a *C*_a_ of 100 μmol CO_2_ mol^− 1^.

### Anatomical analysis

For the anatomical analysis, approximately 1–2 mm^2^ leaf sections were cut and fixed in FAA (95% ethanol: glacial acetic acid: formalin: distilled water = 10:1:2:7), dehydrated in ethanol series, and embedded in paraffin. After cutting into 6 μm transverse sections with a microtome and mounting on glass, the glass was stained with Safranin O and fast green and then mounted in DPX mounting medium. Images of each section were obtained with a light microscope (BX 53, Olympus) with a CCD camera (eXcope X3, DIX, Korea). Leaf thickness (*T*_L_), mesophyll thickness (*T*_m_), leaf density (*D*_L_), and the volume fraction of intercellular air space (*f*_ias_) were measured and/or calculated from at least 5 sections from four different leaves, and at least 5 different fields of view were observed for a given section of images. *D*_L_ and *f*_ias_ were calculated as:

7 $$ {D}_{\mathrm{L}}=\frac{M_{\mathrm{A}}}{T_{\mathrm{L}}} $$

8 $$ {f}_{\mathrm{ias}}=1-\frac{\Sigma {S}_{\mathrm{m}}}{T_{\mathrm{m}}\ \mathrm{W}} $$

where *M*_A_ is the specific leaf weight (g m^− 2^), Σ*S*_m_ is the total sectional area of mesophyll cells, and W is the width of the section.

For the transmission electron microscope (TEM) analysis, approximately 1–2 mm^2^ leaf sections were cut from the middle of newly expanded leaves using two razor blades, fixed in 2.5% glutaraldehyde (0.1 mol L^− 1^ phosphate buffer, pH 7.0) and postfixed with 2% osmium tetroxide. Specimens were dehydrated in a graded acetone series and embedded in Epon 812. Ultrathin cross-sections of 90 nm for transmission electron microscopy (TEM) were cut with a Power Tome-XL ultramicrotome, stained with 2% uranyl acetate, and examined with an H-7650 transmission electron microscope. For each sample, 15 cross-sections were chosen to measure mesophyll cell wall thickness (*T*_mc_) and total length of the mesophyll cells (*L*_mes_) and chloroplasts (*L*_ch_) facing the intercellular air space. At least 40 chloroplasts from TEM were observed to measure the chloroplast traits, including chloroplast length (*L*_c_), chloroplast thickness (*T*_c_), chloroplast section area (*Sec*_c_), distance between two neighbor chloroplasts (Δ*L*_chl_), and chloroplast distance from the cell wall (Δ*L*_cyt_). The surface area of mesophyll cells to the intercellular air-spaces (*S*_mes_), the surface area of chloroplasts exposed to intercellular airspace (*S*_c_), the chloroplast surface area (*Sur*_c_) and volume (*Vol*_c_) were calculated by using the following formula:

9 $$ {S}_{\mathrm{mes}}=\frac{L_{\mathrm{mes}}}{\mathrm{W}}\mathrm{F} $$

10 $$ {S}_{\mathrm{c}}=\frac{L_{\mathrm{c}\mathrm{h}}}{L_{\mathrm{mes}}}{S}_{\mathrm{mes}} $$

where W is the width of the measured section, and F is the curvature correction factor and taken as 1.55 [40].

11 $$ {Sur}_{\mathrm{c}}=4\times \uppi \times {\left(\mathrm{a}\times {\mathrm{b}}^2\right)}^{2/3} $$

12 $$ {Vol}_{\mathrm{c}}=\left(4/3\right)\times \uppi \times \left(\mathrm{a}\times {\mathrm{b}}^2\right) $$

where a = *L*_c_/2, and b = *T*_c_/2.

The chloroplast number per mesophyll cell (*N*_c_) was determined according to the method of Pyke [41]. Briefly, the leaves were cut into 1–5 mm widths with a scalpel or razor blade, submerged in 3.5% (v/v) glutaraldehyde in a tube and kept in the dark at room temperature for 1 h. The glutaraldehyde solution was then replaced with 0.1 M Na-EDTA (pH 9), and the leaf discs were heat-blocked at 60 °C for 12 h and incubated overnight in the dark at 4 °C. To view chloroplasts in individual cells, a piece of tissue was removed from the tube with fine forceps and placed on a microscope slide in a drop of water. A scalpel handle was used to tap and macerate the tissue fairly vigorously, and a Leica DM2700 M microscope with DIC/Nomarski optics was used to image and count chloroplast numbers with changing focus to avoid duplicate and uncounted chloroplasts (Fig. S3).

### The qualification of the anatomical limitations of mesophyll conductance

The one-dimensional gas diffusion model of Tomas et al. [16] was applied in our present study to determine the anatomical limitations of mesophyll conductance, which was given as:

13 $$ {g}_m=\frac{1}{\frac{1}{g_{\mathrm{ias}}}+\frac{{\mathrm{RT}}_k}{H\bullet {g}_{\mathrm{liq}}}} $$

where *g*_ias_ and *g*_liq_ are the gas phase conductance and liquid phase conductance, respectively. R is the gas constant (8.31 Pa m^3^ K^− 1^ mol ^− 1^), *T*_k_ is the absolute temperature, and *H* is Henry’s law constant (2943.3 Pa m^3^ K^− 1^ mol ^− 1^ for CO_2_). The *g*_ias_, was calculated as:

14 $$ {g}_{ias}=\frac{1}{r_{ias}}=\frac{D_a\bullet {f}_{ias}}{\mathit{\Delta }{L}_{ias}\bullet \varsigma } $$

where *r*_ias_ is the resistance of the gas phase to CO_2_, *D*_a_ is the diffusion coefficient for CO_2_ in the gas phase and is set to 1.51 ***×*** 10^− 5^ m^2^ s^− 1^ at 25 °C, *f*_ias_ is the volume fraction of intercellular air space, Δ*L*_ias_ was taken as half of the mesophyll thickness, and ς is the diffusion path tortuosity (1.57 m m^− 1^).

The *g*_liq_ was determined by different components in the cell, including the conductance in the cell wall (*g*_cw_), plasma membrane (*g*_pl_), cytosol (*g*_cyt_), chloroplast envelope (*g*_en_), and stroma (*g*_st_). Eventually, *g*_liq_ was calculated as:

15 $$ {g}_{\mathrm{liq}}=\frac{S_c}{\left({r}_{cw}+{r}_{pl}+{r}_{cyt}+{r}_{en}+{r}_{st}\right)} $$

where *r*_cw_, *r*_pl_, *r*_cyt_, *r*_en_, *r*_st_ are the reciprocal terms of *g*_cw_, *g*_pl_, *g*_cyt_, *g*_en_ and *g*_st_, respectively. We used an estimate of 0.0035 m s^− 1^ for the *g*_pl_ and *g*_en_ as Tomas et al. [16] suggested. In addition, *g*_cw_, *g*_cyt_, and *g*_st_ were calculated as:

16 $$ {g}_i=\frac{1}{r_i}=\frac{r_{\mathrm{f},\mathrm{i}}\bullet {D}_{\mathrm{w}}\bullet {p}_{\mathrm{i}}}{\Delta  {L}_{\mathrm{i}}} $$

where i stands for cell wall, cytosol, or stroma conductance. *r*_f,i_ accounted for the reduction in the aqueous phase diffusion coefficient for CO_2_ (*D*_w_, 1.79 ***×*** 10^− 9^ m^2^ s^− 1^ at 25 °C) and was taken as 1.0 for cell walls and 0.3 for cytosol and stroma, respectively. *p*_i_ was the effective porosity (m^3^ m^− 3^) and was taken as 1.0 for the cytosol and stroma and 0.28 for the cell walls. Δ*L*_i_ (m) is the diffusion path length in the corresponding component of the diffusion pathway.

The proportion of *g*_m_ determined by limited gas-phase conductance (*l*_ias_) was calculated as:

17 $$ {l}_{\mathrm{ias}}=\frac{g_{\mathrm{m}}}{g_{\mathrm{ias}}} $$

The share of *g*_m_ by different components of the cellular phase conductance (*l*_i_) was determined as:

18 $$ {l}_{\mathrm{i}}=\frac{g_{\mathrm{m}}}{g_{\mathrm{i}}\ {S}_{\mathrm{m}}} $$

### Statistical analysis

One-way ANOVA was applied to assess the differences in each parameter among the treatments with the SPSS 16.0 statistical software package. Significant differences (*P* < 0.05) among treatments are indicated by different letters using the least significant difference test.

## Supplementary Information

**Additional file 1:** **Figure S1.** Representative light micrographs (*A* ~ F; scale bar = 200 μm) and transmission electron micrographs (*G ~ L*; scale bar = 5 μm; *M ~ R*, scale bar = 1 μm) of rice leaves supplied with NH_4_^+^ (AN) or NO_3_^−^ (NN) under 3 different amounts, low N (0.71 mM, LAN and LNN), intermediate N (2.86 mM, MAN and MNN), and high N (7.14 mM, HAN and HNN). UEP, upper epidermis; LEP, lower epidermis; V, vascular bundle; CP, chloroplast; CW, cell wall; SG, starch grain; OG, osmiophilic globule. **Figure S2.** The relationship between mesophyll diffusion conductance (*g*_m_) measured with the Harley et al. method and *g*_m_ modeled with anatomical parameters (Eq. 13–16). Values are means ± SD of four replicates. The data were fitted by linear regression. Broken lines correspond to the 1:1 relationship. **Figure S3.** Differential interference contrast image of chloroplasts in mesophyll cells separated from leaves. Leaves were cut into small pieces and fixed with 3.5% glutaraldehyde, and the mesophyll cells were individually dispersed on the glass plate and observed by microscopy. The red circles in the figure indicate individual mesophyll cells, and the chloroplast numbers therein were counted; the arrows indicate that the mesophyll cells did not separate efficiently. Bars = 20 μm.

## Data Availability

The datasets used and analyzed during the current study are available from the corresponding author on reasonable request.

## Abbreviations

*C*_C_: Chloroplast CO_2_ concentration
*C*_i_: Intercellular CO_2_ concentration
*D*_L_: Leaf density
*g*_m_: Mesophyll conductance
*g*_s_: Stomatal conductance
HAN: High NH_4_^+^
HNN: High NO_3_^−^
LAN: Low NH_4_^+^
*L*_c_: Chloroplast length
LNN: Low NO_3_^−^
*M*_A_: Specific leaf weight
MAN: Intermediate NH_4_^+^
MNN: Intermediate NO_3_^−^
*N*_c_: Chloroplasts number per mesophyll cell
*N*_L_: Leaf nitrogen content
*P*_n_: Net photosynthetic rate
PNUE: Photosynthetic nitrogen use efficiency
*S*_c_: Chloroplast surface area facing intercellular air spaces
*Sec*_c_: Chloroplasts section area
*S*_mes_: The surface area of mesophyll cells to the intercellular air-spaces
*Sur*_c_: Chloroplast surface area
*Vol*_c_: Chloroplast volume
*T*_c_: Chloroplast thickness
*T*_L_: Leaf thickness
*T*_m_: Mesophyll thickness
*T*_mc_: Mesophyll cell wall thickness
