# Supplementary material for: Anatomically induced changes in rice leaf mesophyll conductance explain the variation in photosynthetic nitrogen use efficiency under contrasting nitrogen supply
Source: BMC Plant Biol. 2020 Nov 18;20:527. doi: 10.1186/s12870-020-02731-7 (PMC7672947; doi:10.1186/s12870-020-02731-7)
Supplement: Supplementary file 1 — Additional file 1: Figure S1. Representative light micrographs (A ~ F; scale bar = 200 μm) and transmission electron micrographs (G ~ L; scale bar = 5 μm; M ~ R, scale bar = 1 μm) of rice leaves supplied with NH4+ (AN) or NO3− (NN) under 3 different amounts, low N (0.71 mM, LAN and LNN), intermediate N (2.86 mM, MAN and MNN), and high N (7.14 mM, HAN and HNN). UEP, upper epidermis; LEP, lower epidermis; V, vascular bundle; CP, chloroplast; CW, cell wall; SG, starch grain; OG, osmiophilic globule. Figure S2. The relationship between mesophyll diffusion conductance (gm) measured with the Harley et al. method and gm modeled with anatomical parameters (Eq. 13–16). Values are means ± SD of four replicates. The data were fitted by linear regression. Broken lines correspond to the 1:1 relationship. Figure S3. Differential interference contrast image of chloroplasts in mesophyll cells separated from leaves. Leaves were cut into small pieces and fixed with 3.5% glutaraldehyde, and the mesophyll cells were individually dispersed on the glass plate and observed by microscopy. The red circles in the figure indicate individual mesophyll cells, and the chloroplast numbers therein were counted; the arrows indicate that the mesophyll cells did not separate efficiently. Bars = 20 μm. [file 12870_2020_2731_MOESM1_ESM.docx]

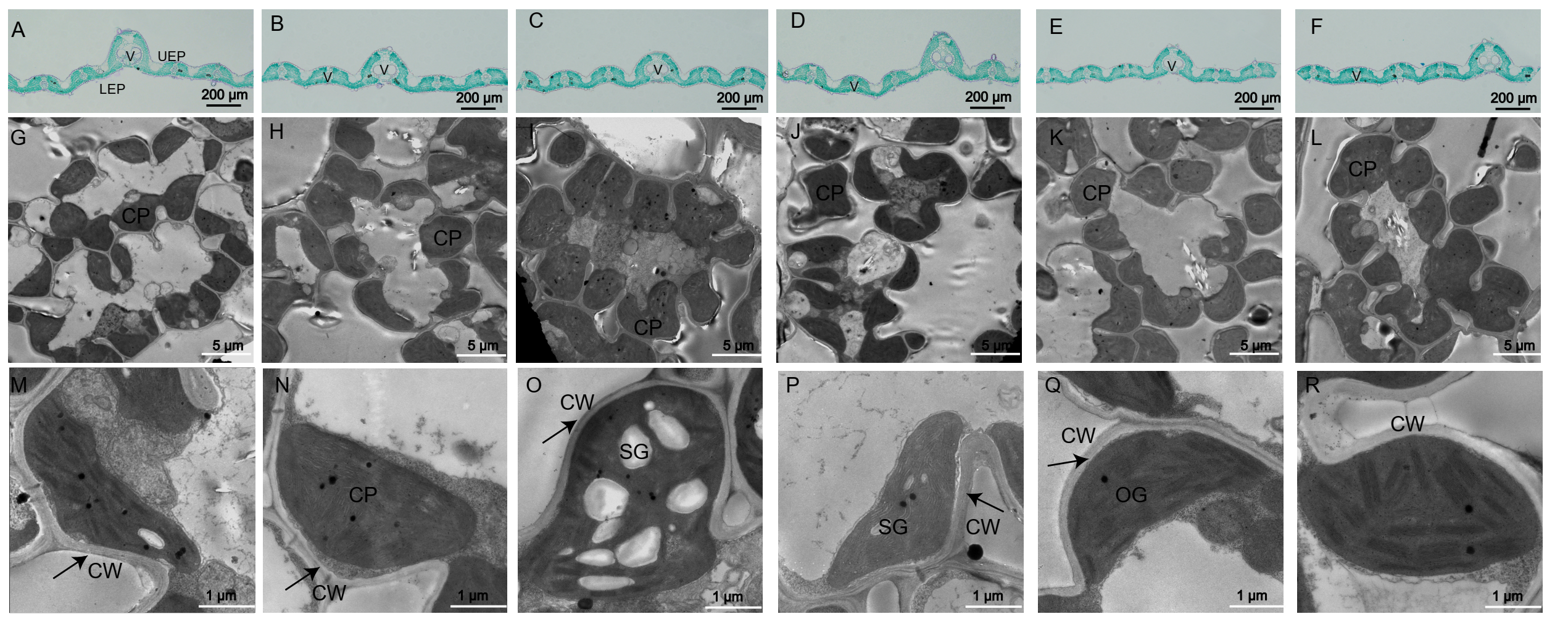
Fig. S1 Representative light micrographs (A~F; scale bar = 200 μm) and transmission electron micrographs (G~L; scale bar = 5 μm; M~R, scale bar = 1 μm) of rice leaves supplied with NH_4_^+^ (AN) or NO_3_^-^ (NN) under 3 different amounts, low N (0.71 mM, LAN and LNN), intermediate N (2.86 mM, MAN and MNN), and high N (7.14 mM, HAN and HNN). UEP, upper epidermis; LEP, lower epidermis; V, vascular bundle; CP, chloroplast; CW, cell wall; SG, starch grain; OG, osmiophilic globule.


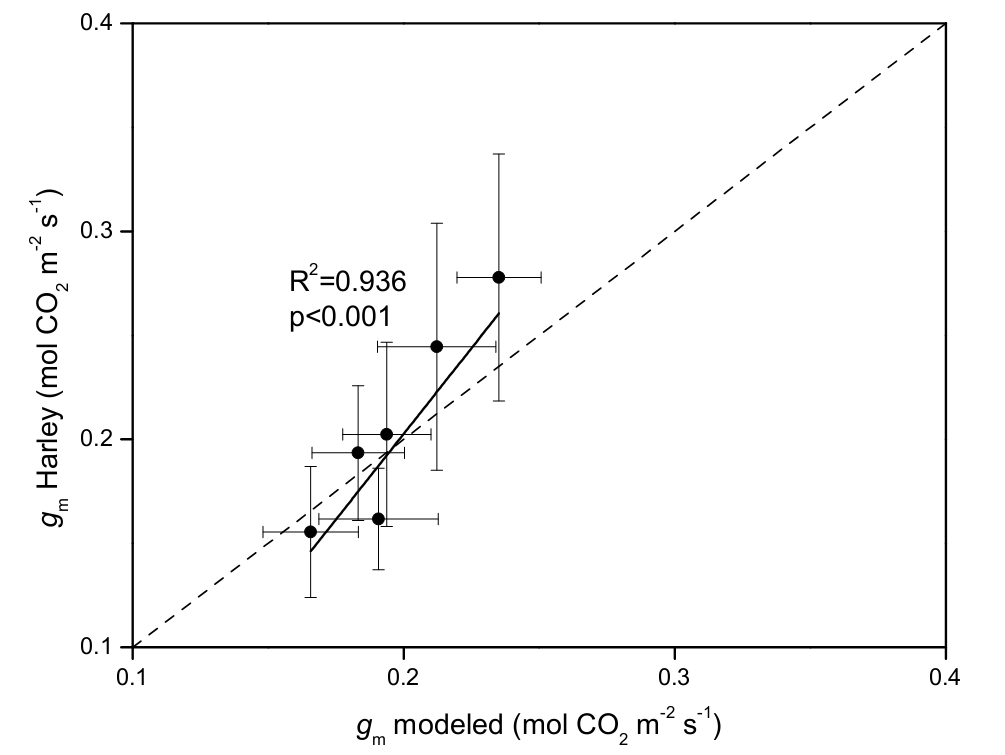
Fig. S2 The relationship between mesophyll diffusion conductance (*g*_m_) measured with the Harley et al. method and *g*_m_ modeled with anatomical parameters (Eqn 13–16). Values are means ± SD of four replicates. The data were fitted by linear regression. Broken lines correspond to the 1:1 relationship.


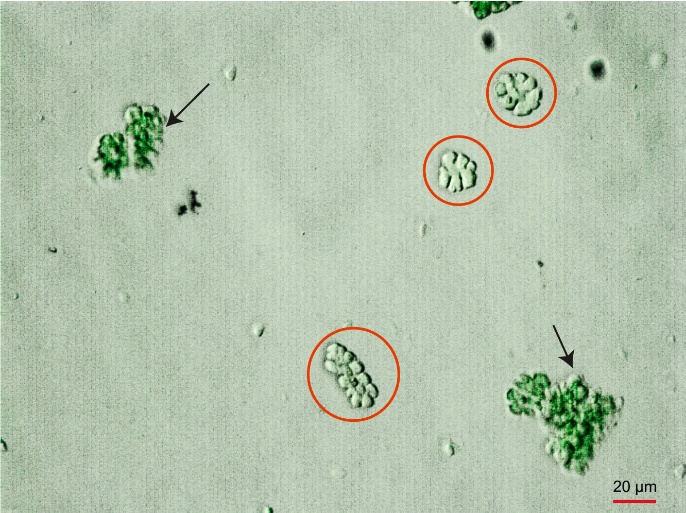
 Fig. S3 Differential interference contrast image of chloroplasts in mesophyll cells separated from leaves. Leaves were cut into small pieces and fixed with 3.5% glutaraldehyde, and the mesophyll cells were individually dispersed on the glass plate and observed by microscopy. The red circles in the figure indicate individual mesophyll cells, and the chloroplast numbers therein were counted; the arrows indicate that the mesophyll cells did not separate efficiently. Bars = 20 μm.
